# Invasive treatment of persistent postoperative chylothorax secondary to thoracic duct variation injury: Two case reports and literature review

**DOI:** 10.1097/MD.0000000000031383

**Published:** 2022-10-28

**Authors:** Qiwei Yang, Xu Bai, Han Bao, Yukang Li, Wanfu Men, Ling Lv, Zhenghua Liu, Xiangjun Han, Wenya Li

**Affiliations:** a Department of Thorax, The First Hospital of China Medical University, Shenyang, Liaoning, China; b Department of Interventional Radiology, The First Hospital of China Medical University, Shenyang, Liaoning, China.

**Keywords:** chylothorax, complication, lymphangiography, pulmonary resection, variation

## Abstract

Postoperative chylothorax is a rare complication after pulmonary resection. Thoracic duct variations may play a key role in postoperative chylothorax occurrence and make treatment difficult. No studies in the literature have reported the successful treatment of chylothorax second to thoracic duct variation by lipiodol-based lymphangiography.

**Patient concerns:**

A 63-year-old male and a 28-year-old female with primary lung adenocarcinoma were treated by video-assisted thoracoscopic cancer resection, and suffered postoperative chylothorax. Conservative treatment was ineffective, including nil per os, persistent thoracic drainage, fatty food restriction, and somatostatin administration.

**Diagnosis:**

Postoperative chylothorax.

**Interventions:**

Patients received lipiodol-based lymphangiography under fluoroscopic guidance. Iatrogenic injuries were identified at thoracic duct variations, including an additional channel in case 1 and the lymphatic plexus instead of the thoracic duct in case 2.

**Outcomes:**

Thoracic duct variations were identified by lipiodol-based lymphangiography, and postoperative chylothorax was successfully treated by lipiodol embolizing effect.

**Lessons:**

Thoracic duct variations should be considered after the failure of conservative treatment for postoperative chylothorax secondary to pulmonary resection. Lipiodol-based lymphangiography is valuable for identifying the thoracic duct variations and embolizing chylous leakage.

## 1. Introduction

Chylothorax is a relatively rare complication after pulmonary resection, with an incidence of 1.4% to 2.3%.^[1,2]^ It usually occurs in the lymphadenectomy stage, and lymphatic fluid leaks out from the thoracic duct and its branches due to iatrogenic injury. As a result of the low incidence, factors affecting chylothorax after pulmonary surgery are still under investigation. From our perspective, anatomic abnormities of lymphatic circulation may play a potential role in chylothorax occurrence. Lipiodol-based lymphangiography has been investigated as an effective imaging method to identify lymphatic abnormities. However, to the best of our knowledge, there are no previous reports of successfully treatment of persistent postoperative chylothorax secondary to thoracic duct variation injury.

Herein, we present 2 specific cases of chylothorax after video-assisted thoracoscopic surgery for lung cancer and demonstrate anatomic abnormities in thoracic duct by percutaneous groin intranodal lymphangiography, all cases were successfully treated by the lipiodol-based lymphangiography. Moreover, postoperative chylothorax in patients with thoracic duct variations are summarized by an in-depth review of the available literature.

## 2. Cases report

### 2.1. Case 1

A 63-year-old male presented to our hospital with a mild cough for 1 week. The medical history included a 40-pack-year smoking history and a thyroidectomy for thyroid cancer 2 years before with a complication of chylous leakage in the neck. Chest computed tomography (CT) examination revealed a pulmonary nodule with a diameter of 2.1 × 1.7 cm in the right upper lobe, and the hilar and mediastinal lymph nodes were not swollen. A tumor marker of carcinoembryonic antigen increased to 5.41 ng/mL while other blood biochemical indices were unremarkable. The patient underwent an upper lobe resection of the right lung and mediastinal lymph node dissection by video-assisted thoracoscopy. The postoperative pathological results revealed that the nodule was primary lung adenocarcinoma, and the stage was pT1cN0M0 IA3 according to the international staging system. On the first day after oral intake, 850 mL of chylous fluid was drained through the thoracic drainage tube. After CT examination and a positive laboratory chylous test, chylothorax was diagnosed. The patient underwent conservative treatment, including nil per os, persistent thoracic drainage, fatty food restriction, and somatostatin administration. After 3 weeks of treatment, chylothorax was still not under control. Finally, percutaneous groin intranodal lymphangiography was performed to identify and embolize the lymphatic leakage site.

**Figure 1.**
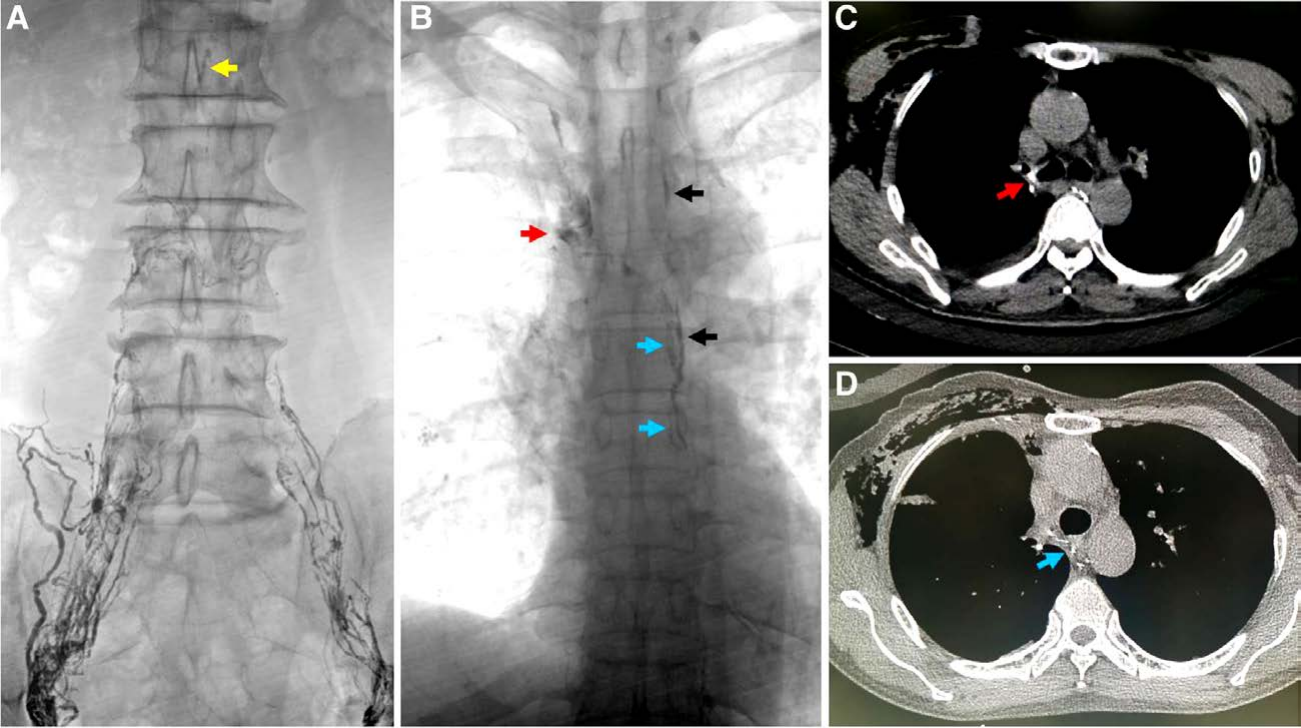
Lipiodol-based lymphangiography and CT showing an additional thoracic duct and leakage from the abnormal vessels. The additional lymphatic channel paralleled to the thoracic duct. Chylous leakage was found at the right pulmonary hilum. (A) Abdominal lymphatic channels. (B) Thoracic lymphatic channels. (C) Chylous leakage at the right pulmonary hilum. (D) Additional thoracic duct. (yellow arrow: chylous cistern; red arrow: leakage site; black arrow: thoracic duct; blue arrow: additional thoracic duct). CT = computed tomography.

Lymphangiography was conducted in bilateral inguinal lymph node access with a 22G needle (Neff percutaneous access set, Cook Medical Company) puncture under ultrasound guidance (IU22, Phillip Company, Netherlands). Nineteen milliliters of lipiodol (480 mg I/mL, Hengrui Company, China) was injected at a rate of 10 mL/h under fluoroscopy guidance (Artis Zee ceiling, Siemens, Germany). The lipiodol dose was determined from the beginning of lipiodol injection via lymph node access to the lymphatic leakage clearly demonstrated. The thoracic duct was demonstrated clearly at the left of the spine, and no injury was found. In addition, an abnormal lymphatic channel was found parallel to the thoracic duct and moved to the right side of the spine across the fourth thoracic vertebra, and lipiodol leaked from the abnormal lymphatic vessel into the thoracic cavity at the right lung hilum. A repeated CT examination also confirmed leakage at the right lung hilum (Fig. 1).

On the first day after lymphangiography, chest drainage decreased to less than 50 mL/d and vanished on postoperative day (POD) 4. Neither cancer nor chylothorax recurred at the 7-month follow-up.

### 2.2. Case 2

A 28-year-old female presented to our hospital with a diagnosis of chylothorax and persistent thoracic drainage of 200 mL/d for 49 days. Her medical history included a left primary lung adenocarcinoma resection using a video-assisted thoracoscope in another medical institution (the cancer stage of pT1bN0M0 IA2). Chylothorax occurred on POD 1 and was treated by a conservative strategy, including nil per os, persistent thoracic drainage, fatty food restriction, somatostatin administration, and pleurodesis with hypertonic glucose injection in another hospital. After the failure of conservative treatment, lymphangiography was recommended in our institution.

**Figure 2.**
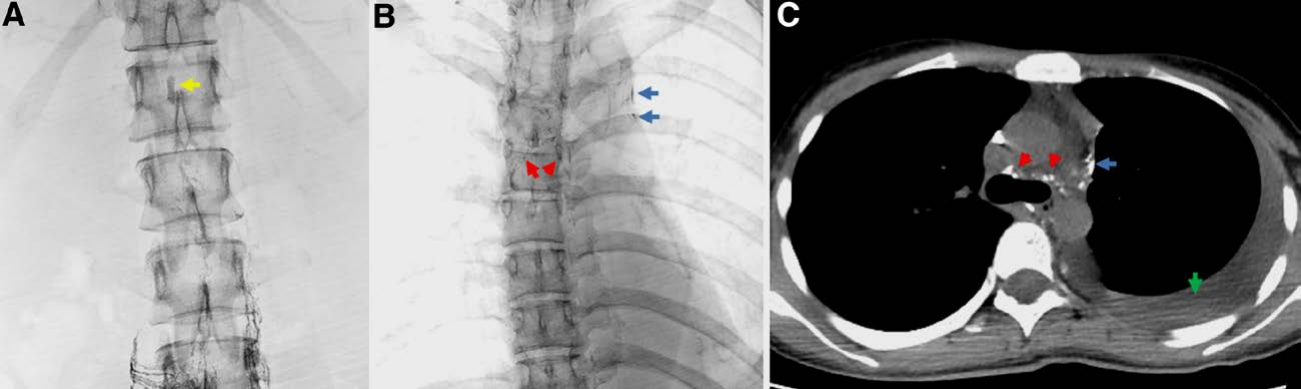
Lipiodol-based lymphangiography and CT showing the lymphatic plexus instead of the thoracic duct and leakage from the vessels. Lymphangiography under fluoroscopy showed a capillary network instead of the thoracic duct and leakage on the left side of the middle mediastinum. (A) Abdominal lymphatic channels showed a chylous cistern without thoracic duct. (B) thoracic lymphatic channels revealed the lymphatic plexus instead of the thoracic duct. (C) CT image at the chylous leakage level. (yellow arrow: chylous cistern; red arrow: plexus thoracic duct; blue arrow: leakage site; green arrow: chylothorax). CT = computed tomography.

Lymphangiography was performed using the same method as in case 1. Lipiodol (13 mL) was injected under fluoroscopy guidance. The thoracic duct was not demonstrated. Instead, the lymphatic vessel was shown to be a capillary network at the level of the lung hilum. Fortunately, the lymphatic leakage site was found on the left side of the middle mediastinum, and a repeated CT examination in the following day also confirmed leakage on the left side of the middle mediastinum (Fig. 2).

On the first day after lymphangiography, chest drainage was reduced to 140 mL/d and vanished on POD 30. Neither cancer nor chylothorax recurred at the 3 month follow-up.

## 3. Consent for publication

Written informed consents were obtained from patients for publication of this case report and accompanying images. Institutional review board approval was not required at our institution for case report.

## 4. Discussion

Chylothorax is an uncommon but potentially severe complication secondary to thoracic surgical procedures. It is known that the incidence of up to 3% after esophageal cancer resections is higher than that of 0.3% to 2.3% after pulmonary cancer resection.^[3,4]^ The anatomic distribution of thoracic duct more adjacent to the esophagus may play a key role. From this point, we hypothesize that anatomic variation in lymphatic vessels in the thorax also plays a partial role in chylothorax occurrence after pulmonary cancer resection. In the present study, we demonstrated 2 cases of chylothorax secondary to pulmonary adenocarcinoma resection using a video-assisted thoracoscopy. We found that the intragenic damaged thoracic ducts were anatomically malformed.

The normal thoracic duct originates from the cisterna chyli at the level of L1–L2, passes through the aortic hiatus of the diaphragm and enters the thoracic cavity along the aorta, esophagus, and azygos vein. Then, the thoracic duct crosses the midline from right to left at the T5–T6 level, rises above the left brachiocephalic vein, and turns down to the left subclavian vein at the confluence of the jugular vein.^[5,6]^ However, the normal anatomy of the thoracic duct only accounts for 50% of individuals, and in the remaining 50%, the thoracic duct anatomy may vary widely.^[7]^ Complicated and multiple anatomical variations in the thoracic duct make it difficult for surgeons to protect lymphatic vessels during thoracic procedures, which also increases the possibility of chylothorax due to intragenic injury of the various thoracic ducts and their tributaries.^[8]^ The thoracic ducts in our cases showed variant performance in lymphangiography. An additional thick lymphatic channel from the lumbar trunk to the thorax was found parallel to the thoracic duct in case 1, and a lymphatic flow plexus instead of the thoracic duct was found in case 2. Interestingly, all iatrogenic lymphatic injuries were located at the variant lymphatic channels, and chylothorax occurred as a complication secondary to pulmonary resection.

Since the anatomic distribution of thoracic duct plays a crucial role in chylothorax during thoracic surgery, identifying the thoracic duct would be of great value before surgery for cancer or reoperation for chylous leakage. According to our literature review, few methods are employed for lymphatic leak identification. The first step is feeding the patient a high-fat diet 2 or 3 hours before thoracic duct ligation, and the increased chylous flow from damaged lymphatic channels makes the surgeon correctly find the leakage location.^[9]^ Although this method is commonly used, it cannot demonstrate the whole thoracic duct morphology and distribution before cancer surgery. The second method is T2-weighted magnetic resonance or magnetic resonance lymphangiography by injecting gadolinium-based contrast material into groin lymph nodes,^[10]^ which is efficient for demonstrating thoracic lymphatic channels without surgical invasiveness and has been used for evaluating lymphatic malformation and abnormities even in children.^[11,12]^ The third is percutaneous fluoroscopic lymphangiography based on lipiodol, which can demonstrate whole lymphatic channels and intragenic leakage with a technical success rate of 75% to 100%.^[13]^ Fluoroscopic lymphangiography also plays a therapeutic role for chylothorax with a clinical success rate of 73% to 95%.^[13, 14]^ Due to the inflammatory and embolic reaction of lipiodol, it usually takes a relative long time to treat the chylous leakage. From our perspective, a month maybe needed to observe the treatment effect. Finally, lymphoscintigraphy can also be used to detect lymphatic defects based on single photon emission computed tomography,^[15, 16]^ and the sensitivity and specificity were reported to be 88% and 100%, respectively.^[17]^

For the treatment of iatrogenic chylothorax, conservative measures including nil per os, low-fat diet, parenteral nutrition, somatostatin administration, and pleural drainage, can greatly reduce chylous flow production and promote leakage healing, which is effective in 60% to 90% of cases.^[2,18]^ If conservative treatment fails, surgical measures of thoracic duct ligation should be considered as the alternative choice. However, due to the increased medical risks and economic burden, there is no consensus regarding surgical indications after conservative treatment fails. Liu et al suggested that pleural drainage of over 400 mL on POD 4 was a good candidate for surgery,^[18]^ and Petrella et al recommend that surgical treatment should be chosen for pleural drainage of over 800 mL on POD 5. The clinical success rate of surgical treatment is 85% to 100%.^[9,19]^ Recently, percutaneous lymphangiography, thoracic duct embolization, and sclerotherapy have been reported as new strategies for chylous leakage. These interventions demonstrate effective and minimally invasive features and can be used as alternative choices to surgical treatment with a clinical success rate of 75% to 100%.^[20, 21]^ In additional to all the above treatments, there is another choice of pleurodesis by injecting materials into the pleural cavity, such as tetracycline, bleomycin, and hypertonic glucose.^[3]^

Due to general unfamiliarity regarding lymphatic variation and the low incidence of chylothorax after pulmonary resection, lymphangiography is not commonly conducted before thoracic surgery. Iatrogenic injury to lymphatic vessels usually occurs at the thoracic duct branches, which is different from esophagectomy. Conservative treatment is effective and should be prioritized. If conservative management fails, thoracic duct variation should be suspected and investigated before reoperation. We searched the PubMed database using the key words “chylothorax” AND “thoracic duct variation”. Eight cases from literature review received thoracic surgery and suffered postoperative chylothorax, and the anatomical variations of thoracic duct were identified during the treatment. Table 1 summarizes the thoracic duct variations in patients with postoperative chylothorax. All thoracic duct variations bring great difficulties for further therapy. All cases in Table 1 underwent either long-term treatment and/or 2 surgical thoracic duct ligations. Surgical procedures including 5 prophylactic thoracic duct ligations before chylothorax, 3 therapeutic thoracic duct ligations after chylothorax, and 4 other surgical managements were ineffective in 8 cases before thoracic duct variation confirmation. Our results also revealed that surgical thoracic duct ligation would not have been effective for treating the abnormal thoracic duct in case 1 and the absence of thoracic duct in case 2. Thus, we recommend that percutaneous groin lymph node lymphangiography using lipiodol be prioritized after conservative failure in chylothorax secondary to pulmonary cancer resection. Lipiodol-based lymphangiography is valuable in demonstrating lymphatic morphology and embolizing the leakage, which is also helpful for doctors to conduct thoracic duct ligation.

**Table 1:** Reports of thoracic duct variations in patients with postoperative chylothorax.

Study	Age (yr)/sex	Operation	Interval to onset (d)	Pleural drainage (mL/d)	Thoracic duct morphology	Treatment	Ref
Lary A 2022	69/F	Left lower lobectomy; Left upper lobe wedge resection; Mediastinal lymphadenectomy by limited left thoracotomy	POD 1	1200–1750	Aberrant lymphatic plexus	Conservative treatment (12 d; failure) Lymphangiography (POD4; technical failure) Parietal pleurectomy and talc pleurodesis byre-entry left thoracotomy (POD 8; failure) Surgical TD ligation above diaphragm (POD 39; failure) Left pleural-venous Denver shunt insertion (POD44; failure) Left chest re-exploration (POD 56; failure) Lymphangiography (POD 64; find variation) Radiotherapy (POD88; failure) Lymphangiography and surgical resecting all tissue including left TD ligation below diaphragm (POD 108; success)	^[22]^
Daisuke 2021	66/M	Right upper lobectomy; Hilar mediastinal lymphadenectomy by VATS Lymphatic duct dissection and ligation	POD 1	500	TD flow into right venous angle	Conservative treatment (1 d; failure) Surgical right TD ligation (POD 2; find variation; success)	^[23]^
Ryoma 2020	53/F	Subtotal esophagectomy; 3-field lymphadenectomy via left thoracotomy; Esophagogastrostomy; Left mediastinal pleura partial resection around the tumor; Right TD resection	POD 1	NA	Bilateral TD	Conservative treatment (2 d; failure) Lymphangiography and embolization (POD 6; find variation; failure) Surgical left TD ligation (POD 8; success)	^[24]^
Benoit 2020	60/M	Open lower bilobectomy; Mediastinal lymphadenectomy	POD 1	NA	Bilateral TD	Conservative treatment (11 d; failure) Surgical right TD ligation (POD 12; failure) Lymphangiography (POD 20; find variation) Surgical left TD ligation (POD 22; success)	^[25]^
Tomoyuki 2019	52/M	Esophagectomy; Lymph node dissection via thoracoscopy and laparoscopy; Esophagogastrostomy; Right TD ligation	POD 30	NA	Bilateral TD	Conservative treatment (10 d; failure) MRI (POD40; find variation) Lymphangiography (POD 44; failure) Lymphangiography (POD 59; failure) Pleurodesis with OK-432 (POD75; POD78; success)	^[26]^
Takeshi 2017	72/M	Subtotal esophagectomy; 2-field lymphadenectomy via a right thoracoabdominal approach; Esophagogastrostomy	POD 12	1000	Bilateral TD	Conservative treatment (8 d; failure) Surgical right TD ligation (POD 19; failure) Lymphangiography (POD 24; find variation) Surgical left TD ligation (POD 25; success)	^[27]^
Tetsuya 2016	69/M	Subtotal esophagectomy; 3-field lymphadenectomy via right thoracotomy and laparotomy; Esophagogastrostomy; Right TD ligation and resection	POD 2	2000–2850	Bilateral TD	Conservative treatment (4 d; failure) Lymphangiography (POD 6; find variation) Surgical left TD ligation (POD 9; success)	^[28]^
Shinji 2008	58/M	Esophagectomy; 3-field lymphadenectomy via right thoracotomy; Esophagogastrostomy; Right TD ligation and resection	POD 120	800–1000	Bilateral TD	Conservative treatment (NA; failure) Lymphangiography (NA; find variation) Surgical left TD ligation (NA; success)	^[29]^

F = female, M = male, NA = not available, POD = postoperative day, TD = thoracic duct, VATS = video-assisted thoracoscopic surgery. MRI = Magnetic resonance imaging

In conclusion, chylothorax is an infrequent complication after pulmonary cancer surgery. We reported 2 cases of chylothorax that are difficult to treat. The present cases suggest that thoracic duct variations should be considered after the failure of conservative treatment. Minimally invasive lipiodol-based lymphangiography is helpful to identify the morphology and damage of thoracic duct, which also plays a therapeutic role and should be given more patience before reoperation.

## Acknowledgments

We thank the American Journal Experts for English editing.

## Author contributions

**Conceptualization:** Xiangjun Han, Wenya Li.

**Data curation:** Xu Bai, Han Bao, Yukang Li, Wanfu Men, Ling Lv, Zhenghua Liu.

**Supervision:** Xiangjun Han, Wenya Li.

**Writing – original draft:** Qiwei Yang.

**Writing – original draft:** Xu Bai. QY and XB contributed equally
